# Atypical alert state control in adult patients with ADHD: A pupillometry study

**DOI:** 10.1371/journal.pone.0244662

**Published:** 2020-12-30

**Authors:** Aya Shirama, Toshinobu Takeda, Haruhisa Ohta, Akira Iwanami, Shigenobu Toda, Nobumasa Kato

**Affiliations:** 1 Medical Institute of Developmental Disabilities Research, Showa University, Tokyo, Japan; 2 NTT Communication Science Laboratories, Nippon Telegraph and Telephone Corporation, Kanagawa, Japan; 3 Faculty of Letters Ryukoku University, Kyoto, Japan; 4 Department of Psychiatry, School of Medicine, Showa University, Tokyo, Japan; 5 Department of Psychiatry, Showa University East Hospital, Showa University, Tokyo, Japan; 6 Department of Psychiatry and Behavioral Science, Kanazawa University, Kanazawa, Japan; Istituto di Fisiologia Clinica Consiglio Nazionale delle Ricerche, ITALY

## Abstract

Although behavioral studies have repeatedly demonstrated that individuals with attention-deficit/hyperactivity disorder (ADHD) have deficits in alertness, little is known about its underlying neural basis. It is hypothesized that pupil diameter reflects the firing of norepinephrine (NE) neurons in the locus coeruleus (LC), and that the LC-NE neuromodulatory system for regulating alertness may be dysfunctional in ADHD. To clinically and non-invasively examine this hypothesis, we monitored the kinetics of pupil diameter in response to stimuli and compared them between adults with ADHD (n = 17) and typically developing (TD) adults (n = 23) during an auditory continuous performance task. Individuals in the ADHD group exhibited a significantly larger tonic pupil diameter, and a suppressed stimulus-evoked phasic pupil dilation, compared to those in the TD group. These findings provide support for the idea that the aberrant regulatory control of pupil diameter in adults with ADHD may be consistent with a compromised state of alertness resulting from a hyperactivated LC-NE system.

## Introduction

Attention-deficit/hyperactivity disorder (ADHD) is characterized by inattention, impulsivity, and hyperactivity [1]. While impulsivity and hyperactivity can improve during development, attentional dysfunction often persists even after adolescence and affects adult patients' cognitive performance [2–4]. Some studies have shown that inattentive symptoms are associated with an increased risk for long-term work disability such as unemployment in adults with ADHD [5, 6].

Alertness is one of the specific types of attentional functions compromised in ADHD [7]. Attenuated alertness in individuals with ADHD has been reported when using the Continuous Performance Test (CPT) which requires sustained attention over a prolonged period ([8, 9], but see [10, 11]). A meta-analysis of studies on task performance in adults with ADHD found medium to large effect sizes on omission errors (inattention) and small to medium effect sizes for commission errors (impulsivity) [12]. Alertness comprises an ability to increase the signal-to-noise ratio of neuronal responses to a relevant stimulus, and the ability of implicit control of arousal to fine-tune the ratio [13]. Norepinephrine (NE) is a key attention-regulating neurotransmitter produced primarily in the locus coeruleus (LC). According to the adaptive gain theory proposed by Aston-Jones and Cohen [7], exploitative and exploratory modes of alerting are balanced by two distinct modes of LC activity (phasic and tonic, respectively). Theoretically, when one needs to maintain alertness toward task-relevant stimuli (exploitative mode), performance is optimal under moderate LC activity at the baseline in combination with prominent phasic (short burst) activity. On the other hand, while searching or examining something new or unfamiliar, performance is optimal under elevated levels of LC baseline activity with limited phasic NE release (explorative mode).

Based on this concept, excessive exploratory-mode activity may result in disturbed alertness due to the implicit capture of too much irrelevant information. Thus, the attenuated alertness in ADHD could be attributed to relatively higher LC activity at the baseline than occurs in typically developing (TD) controls. To verify this hypothesis, pupillometry can be utilized as an indirect but non-invasive approach, because pupil diameter reflects LC firing on time [14–17]. Some pupillometry studies revealed that increased tonic pupil diameter was associated with task disengagement and explorative behavior such as attentional set shifting in healthy individuals [15, 18, 19]. In contrast, relatively small tonic pupil diameter was found to be associated with increased stimulus-evoked pupil dilations and optimal performance in an auditory oddball task [20].

Pupillometry has already been employed for some studies of children and adolescents with ADHD and revealed a decreased phasic pupil dilation after stimulus onset during working memory tasks including a N-back task [21–23]. These studies also found that task performance correlates with maximal pupil diameter after stimulus onset [22, 23]. However, there is no previous study that has measured tonic and phasic pupil responses simultaneously to directly address the relationship between them in adult patients with ADHD in a neurocognitive framework of attention [24].

**Fig 1:**
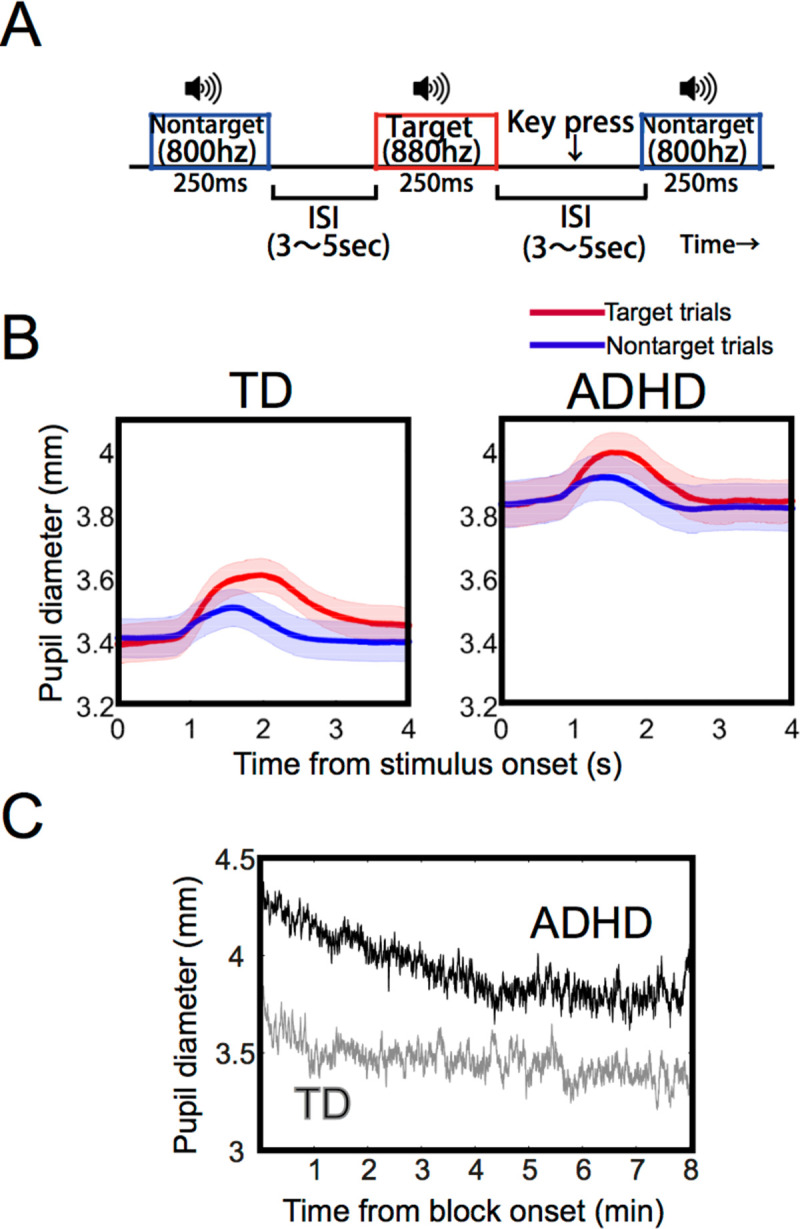
(A) A schematic diagram of the auditory CPT task. The sound was either a nontarget tone (800 Hz, p = .80) or a target tone (880 Hz, p = .20). The participants were asked to push a button when they detected the target tone as quickly and accurately as possible. (B) Alterations in pupil diameter over time during one trial in the TD and the ADHD groups. Red and blue lines indicate the mean values of target and nontarget trials, respectively. Color shaded areas correspond to the standard error of the mean value. (C) Alterations in pupil diameter during one block.

The present study aims to address this deficit by monitoring pupil diameter during an auditory continuous performance test (aCPT, **Fig 1A**) in which the performance of adults with ADHD was found to be worse than that of TD [8, 25]. We hypothesize that pupil regulation in adult ADHD can be characterized by an elevated tonic activity in combination with attenuated task-induced phasic activity during the aCPT, compared to that of TD. We also hypothesize that the attenuated task-induced phasic activity relates to worse performance such as increased reaction times or error rate in the aCPT.

## Results

### Tonic pupil diameter and phasic pupil dilation during the aCPT

**Fig 2:**
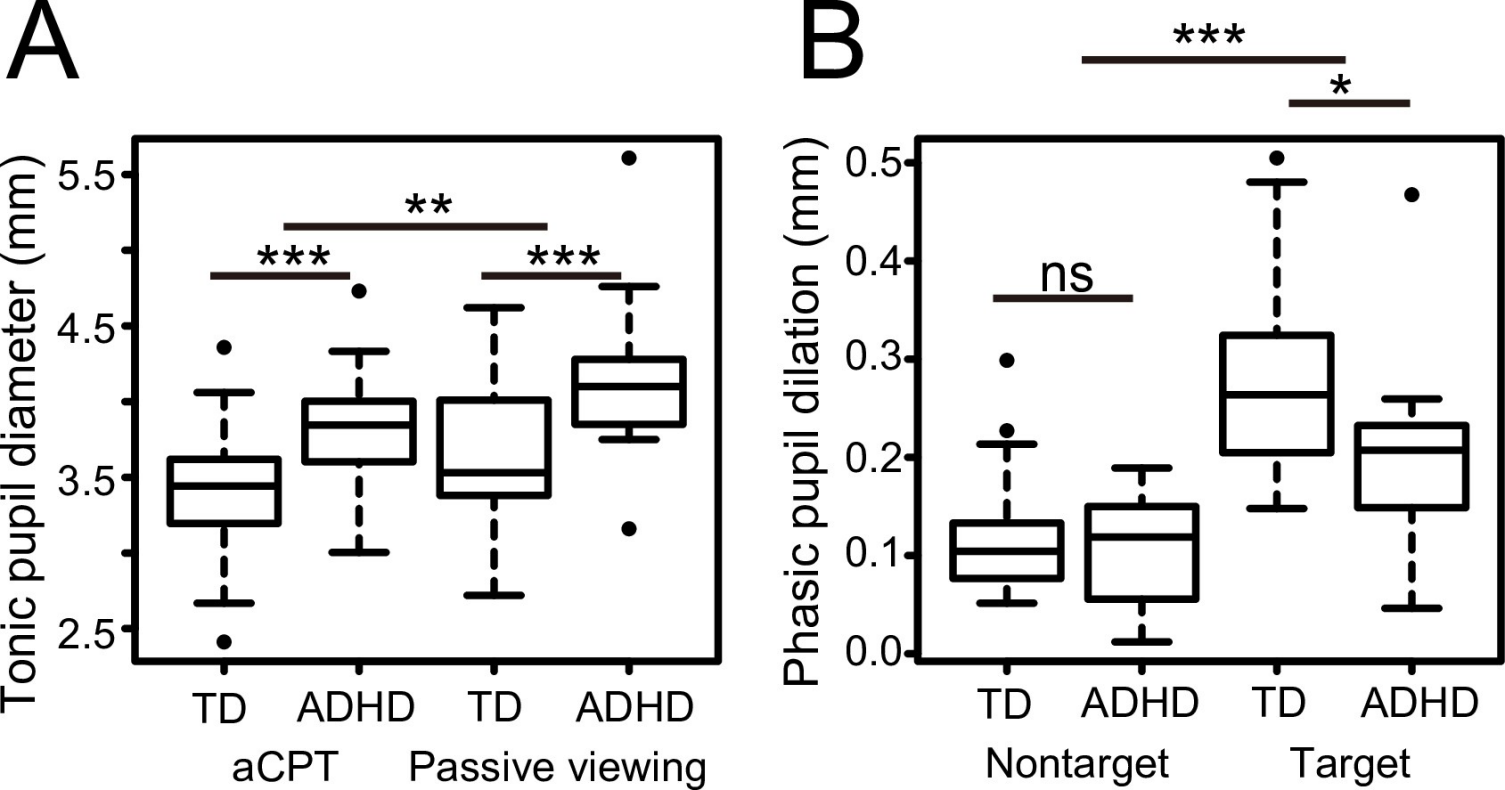
Tonic and phasic pupil diameters in the TD and ADHD groups. (A) Tonic pupil diameter during the passive-viewing condition and the auditory CPT. To estimate tonic pupil diameter, we calculated an average pupil diameter in the period from -1 to 0 s before the stimulus presentation. We calculated phasic pupil dilation during one trial by detecting pupil diameter maxima in a time window between 4 s after stimulus (target, nontarget) onset. (B) Maximum pupil diameter in response to the target and the nontarget tones. These were assessed for statistically significant differences with a mixed-model ANCOVA or ANOVA. *p < .05, **p < .001, ***p < .0001, ns, not significant.

First, we examined if there were differences in tonic pupil diameter and phasic pupil dilation during the task between the two groups. Tonic pupil diameter indicates prestimulus baseline pupil diameter. On the other hand, phasic pupil dilation indicates a difference between a peak pupil diameter after stimulus onset and prestimulus baseline pupil diameter. **Fig 1B** illustrates mean pupil changes over time from the onset of a tone presentation. Although tonic pupil diameter gradually decreased during one block (**Fig 1C**), as **Fig 1B** shows (see also **Fig 2A**), the ADHD group exhibited increased tonic pupil diameter compared to the TD group both in the aCPT (mean 3.39 mm (TD), 3.84 mm (ADHD)) and the passive-viewing task (mean 3.63 mm (TD), 4.16 mm (ADHD)). To test for group differences in tonic pupil diameter, we conducted a repeated-measures analysis of covariance (ANCOVA) with group as a between-participants factor, task as a within-participants factor, and age as a covariate. We used age as a covariate because tonic pupil diameter is thought to become smaller with increasing age [21], and because there was a trend towards a lower age in the ADHD group compared to in the TD group (*p* = .08) in the present study (Table **1**). The analysis showed main effects of group (*F*(1, 36) = 8.1, *p* < .01) and task (*F*(1, 36) = 7.3, *p* < .01), but no interaction between factors (*F*(1, 36) = 0.17, *p* = .68).

**Table 1:** Characteristics of the participants.

	TD	ADHD	*p*-value
N	23 (10M:13F)	17 (8M:9F)	-
AGE years	35.5 ± 1.7	31.7 ± 2.0	*p* = .08
FIQ	102.9 ± 2.9	101.5 ± 3.4	*p* = .38
VIQ	102.6 ± 2.9	102.6 ± 3.3	*p* = .50
PIQ	102.4 ± 3.3	97.9 ± 3.9	*p* = .19
ASRS-J Total	19.7 ± 2.5	42.5 ± 3.3	*p* < .001
ASRS-J Inattention	11.9 ± 1.3	25.4 ± 1.6	*p* < .001
ASRS-J	7.8 ± 1.1	17.3 ± 42.0	*p* < .001
Impulsivity/Hyperactivity			

FIQ: full-scale intellectual quotient, VIQ: verbal intellectual quotient, PIQ: performance intellectual quotient, ASRS: the Japanese version of the adult ADHD self-report scale, ASRS-J Inattention: ASRS-J inattention symptom score, ASRS-J Impulsivity/Hyperactivity: ASRS-J impulsivity/hyperactivity symptom score. These were assessed for statistically significant differences with Student's t-tests.

Both the TD and ADHD groups had transient dilatory responses in pupil diameter after stimulus onset. We compared the phasic pupil dilation in response to the stimulus tones between groups (mean 0.28 mm (target, TD), 0.12 mm (nontarget, TD), 0.20 mm (target, ADHD), 0.11 mm (nontarget, ADHD)) (**Fig 2B**). A mixed-model analysis of variance (ANOVA) with the factors group and stimulus (target vs. nontarget) demonstrated that there was no main effect of group (*F*(1, 36) = 3.7, *p* = .06), but a significant main effect of stimulus (*F*(1, 36) = 146.0, *p* < .001). Furthermore, there was a significant interaction between group and stimulus (*F*(1, 36) = 9.6, *p* < .005). When the responses were subgrouped according to target vs. nontarget trials, only responses to target trials were significantly different (target, *F*(1, 36) = 6.3, *p* < .05; nontarget, *F*(1, 36) = 0.5, *p* = .48). Thus, target tones evoked a greater phasic pupil dilation than nontarget tones as previously reported [15]. In the ADHD group, a phasic pupil response to targets was attenuated compared to that of the TD group.

We also examined the possibility that the ADHD group had reduced phasic pupil dilation simply due to any limitations in pupillary responses caused by elevated tonic pupil diameter. We examined whether there was a linear relationship between phasic pupil dilation and tonic pupil size using regression analysis. We found that tonic pupil diameter had no significant predictive power for phasic pupil dilation (target trials: adjusted *R*^2^ = -.006, *p* = .38, nontarget trials: adjusted *R*^2^ = -.027, *p* = .90).

### Comparison of task performance between groups

**Fig 3:**
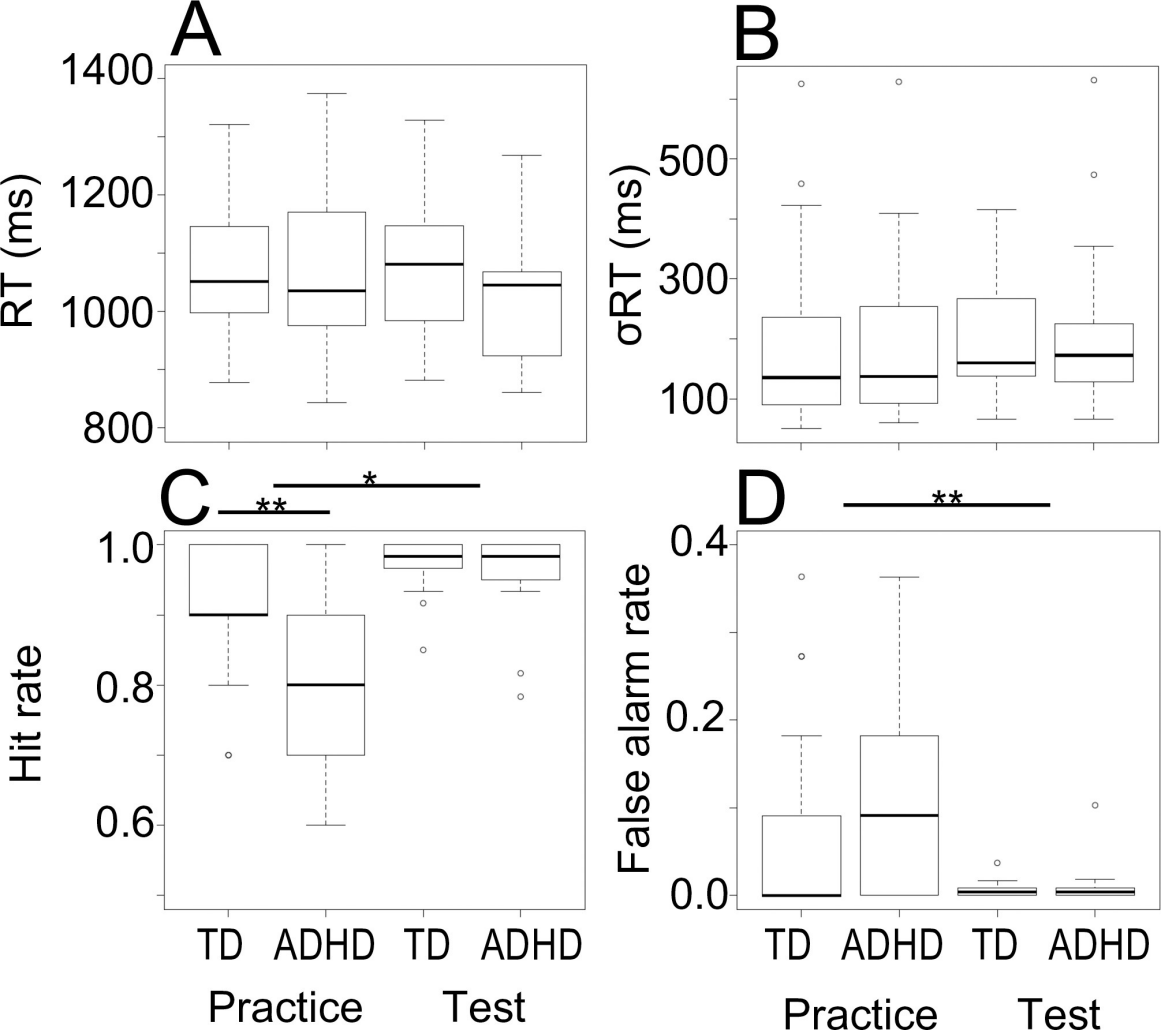
Behavioral performance on the aCPT across group (TD, ADHD) and phase (practice, test). (A) RT (ms), (B)σRT (ms). (C) Hit rate. (D) False alarm rate. σRT: reaction time variability (standard deviation), hit rate: the probability of correctly detecting a target on target trials, false alarm rate: the probability of falsely detecting a target on nontarget trials. These were assessed for statistically significant differences with a mixed-model ANOVA. *p < .05, **p < .001.

Next, we evaluated behavioral variables (RT: reaction time, σRT: reaction time variability, hit rate, false alarm rate). The behavioral variables represent the speed and accuracy of the button press (the participant’s response). **Fig 3** shows behavioral performance on the aCPT across group (TD, ADHD) and phase (practice, test). As shown in **Fig 3**, although detection accuracy was lower in the ADHD group compared to the TD group in the practice trials (hit rate: .91(TD), .72(ADHD); false alarm rate (a probability of falsely detecting a target on nontarget trials): .08(TD), .19(ADHD),), it reached a ceiling in the test trials (hit rate: .98(TD), .96(ADHD); false alarm rate: .01(TD), .01(ADHD)). To test for group differences in the hit rate, we used a mixed-model ANOVA with the factors group and phase (practice vs. test). This demonstrated that there were main effects of group (*F*(1, 36) = 9.23, *p* < .01) and phase (*F*(1, 36) = 17.19, *p* < .001) with a two-way interaction between these factors ((*F*(1, 36) = 5.97, *p* < .05)). Follow-up ANOVAs revealed that the ADHD group had a significantly lower hit rate compared to the TD group in the practice phase (*F*(1, 36) = 7.91, *p* < .05) but not in the test phase (*F*(1, 36) = 1.0, *p* = .32). The same analysis for the false alarm rate showed no main effect of group (*F*(1, 36) = 2.52, *p* = .12), but a significant main effect of phase (*F*(1, 36) = 15.70, *p* < .001) and no significant two-way interaction between these factors (*F*(1, 36) = 2.44, *p* = .13). The RT and RT variability data were also analyzed by using mixed-model ANOVAs with the factors group and phase. Results showed neither main effects of group (RT: *F*(1, 36) = 0.83, *p* = .38, σRT: *F*(1, 36) = 0.09, *p* = .77), or phase (RT: *F*(1, 36) = 1.22, *p* = .28, σRT: *F*(1, 36) = 0.01, *p* = .93) and that there was no two-way interaction between these factors (RT: *F*(1, 36) = 0.81, *p* = .38, σRT: *F*(1, 36) = 0.05, *p* = .83). Both groups had comparable RTs (practice: 1073.2(TD), 1063.6(ADHD), test: 1068.7 ms (TD), 1026.4 ms (ADHD)) and RT variability (practice: 196.9(TD), 198.9(ADHD), test: 192.6 (TD), 209.1 (ADHD)).

**Fig 4:**
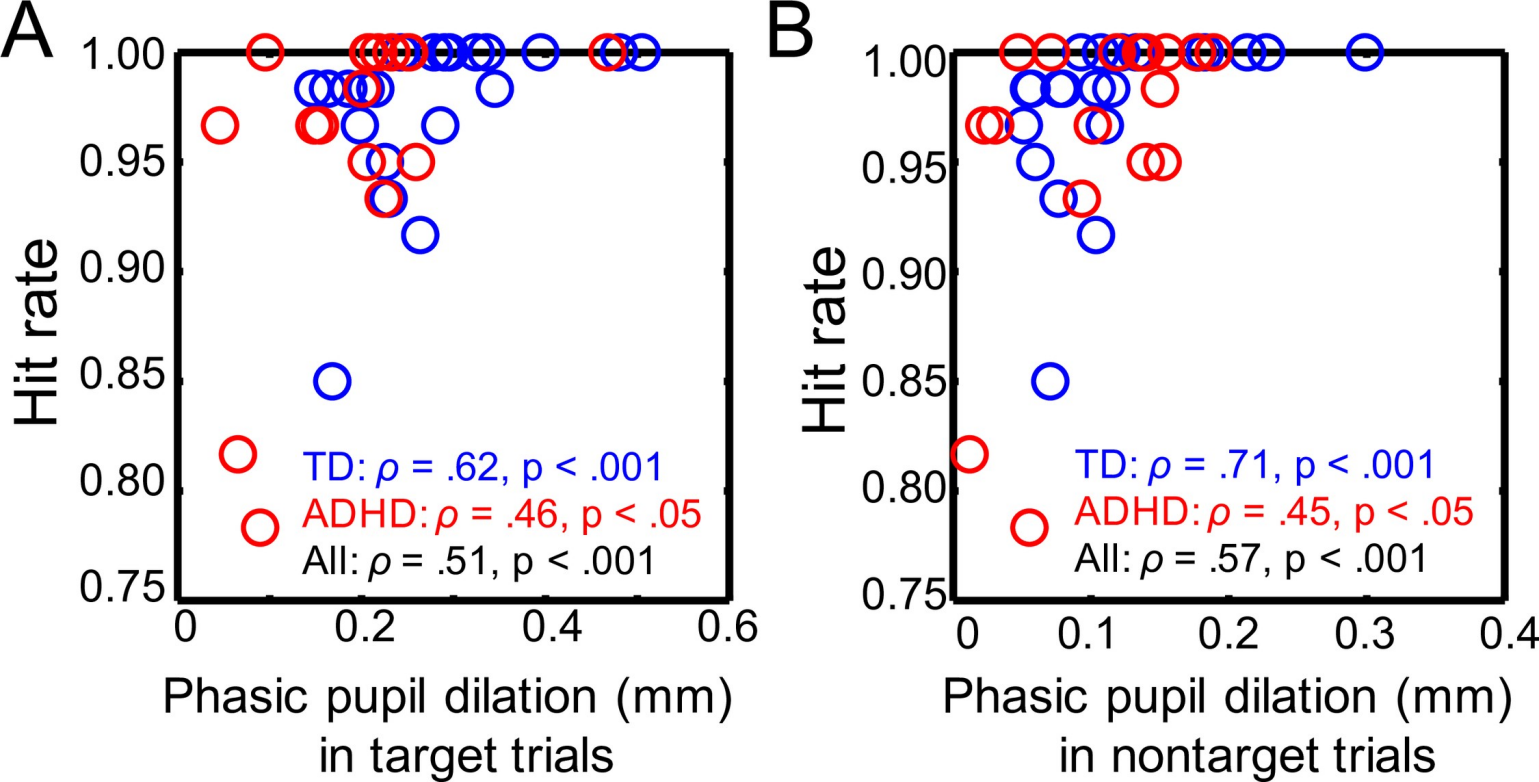
Comparisons of the relationships of task performance with the parameters of pupil diameter between the TD and ADHD groups. Hit rate and phasic pupil dilation in the target trials (A) and in the nontarget trials (B). Hit rate: the probability of correctly detecting a target on target trials.

We also evaluated the relationships between pupillary and behavioral variables by using Spearman’s rank order test. As **Fig 4** shows, phasic pupil dilation was positively correlated with the hit rate in every group, regardless of whether the trials were target or nontarget (target trials: *ρ* = .62, *p* < .001 (TD), *ρ* = .46, *p* < .05 (ADHD)); nontarget trials: *r* = .71, *p* < .0001(TD), r = .45, *p* < .05 (ADHD).

## Discussion

This study aimed to clarify atypical pupillary responses during an aCPT in adults with ADHD. Children and adolescents with ADHD have been previously reported to exhibit decreased phasic pupil dilation after stimulus onset [21–23]. This study revealed more detailed characteristics of pupillary responses in adults with ADHD. First, we confirmed that adults with ADHD had less elevated phasic pupil responses in the aCPT (**Fig 2B**), when compared to those in the TD group. Second, the ADHD group exhibited a significantly increased tonic pupil diameter (**Fig 2A**). To our knowledge, this is the first demonstration of the relationship between tonic and phasic pupil regulation in adult ADHD. These data suggest that in adults, ADHD presents as a relatively hyperarousal state in combination with compromised phasic responses to target stimuli. In addition, the ADHD group consistently showed higher tonic pupil diameter in both the passive viewing task and the aCPT. These differences in pupillary response in adult ADHD are consistent with a compromised alerting condition and hyperactive LC-NE system that would be in line with the adaptive gain theory [24]. On the other hand, the ADHD group showed comparable pupil dilation in nontarget trials compared to that of the TD group (**Fig 2B**). In accordance with basic findings in pupillometry [15], we showed that the magnitude of phasic pupil dilation was larger for targets than for nontargets. It is assumed that the larger pupil response was induced by a motor response which was necessary only in target trials. It should be noted that pupil dilations increase with motor responses [26, 27]. Therefore, the decreased phasic pupil dilation in the ADHD group may reflect dysfunction in cognitive and goal-directed motor processes.

We also found associations between pupillary and behavioral variables; there was a positive correlation between the magnitude of phasic pupil dilation and the hit rate (**Fig 4**). Some earlier studies have shown that attenuated phasic pupil diameter is associated with worse performance in working memory tasks in children and adolescents with ADHD [21–23]. Taken together, these findings suggest that pupillometry may be an efficient method to investigate the neural basis of inattention symptoms in ADHD. Moreover, pupillometry studies of adults and children with ADHD indicate that ADHD could be attributed to the aberrant activity of the LC-NE system and is not only a deficit of the dopaminergic system, as suggested by some authors (for review, see [28]). There is a possibility that the aberrant activities of the dopaminergic and noradrenergic systems differ between individuals and induce interindividual variability in ADHD symptoms. In future research, pupillometry can be a useful tool to noninvasively assess individual differences in the activity of the LC-NE system in real-time.

The results of this study should be considered in light of several study limitations. First, despite our expectation based on the adaptive gain theory that higher NE tone in ADHD should be correlated to lower performance, the ADHD group performed worse in the practice period but not in the test period (**Fig 3C**). Although a meta-analysis of CPT-related studies found small or moderate effect sizes for performance over time [11], it is true that some studies have produced counterevidence [10, 11]. There is a possibility that a simple CPT is too easy to clearly distinguish the difference in task performance between ADHD and TD adults.

Second, pupil diameter can be regulated not only by LC-NE, but also by the cholinergic system [17], but there is currently no direct evidence to support either mechanism. Third, we could not find a linear relationship between tonic pupil diameter and phasic pupil dilation in terms of the between-participant comparison. Some previous studies reported that intraindividual changes in baseline pupil diameter and task-evoked pupil dilations are negatively correlated with each other [15, 20]. It is known that individual differences in pupil diameter relate to factors other than alert state, such as age and intelligence [29]. Although we matched the two groups (ADHD, TD) in terms of their demographic features (Table **1**), these factors can confound between-participant baseline measures. Further research is necessary to establish a reliable method for between-participant comparisons based on pupillary responses.

Fourth, in this study we defined one-day medicine-free as being almost equal to drug-naïve. Although the average half-lives of methylphenidate and atomoxetine are 3.5 and 5 hrs, respectively (www.accessdata.fda.gov), and there are some previous studies that employed similar paradigms [23, 30], it is still possible that these medicines might have already yielded some long-term pharmacological effects that endure for more than a couple of days due to their regular administration. Thus, one-day off medicine may not be sufficient for an individual to be regarded as being genuinely medicine-free.

Fifth, in this study we regarded the passive-viewing condition as a control to be compared with the condition where the subjects were intensively engaging in the aCPT. However, it is plausible that this control condition might also be a preparatory state that requires some alertness, rather than a completely relaxed condition in which the subjects were totally free from the stress of the test [31]. Indeed, the baseline pupil diameter can be changed in a task-related manner under various cognitive conditions such as engagement and memory load [32]. In this scenario, it is possible that what we examined in this study was a comparison between preparatory and task-focusing states, rather than a comparison between a genuine resting state and task-focusing state.

Sixth, it is also possible that the hyperarousal in ADHD reflects a temporal physiological response related to stress in the testing situation. ADHD in adults is associated with increased vulnerability to mental and social stress and may thus increase the risk of chronic stress [33]. A recent study has shown that experimentally-induced stress led to heightened perceived stress and increased salivary cortisol [34]. Investigating the associations between perceived-stress, cortisol level, and tonic pupil diameter is required in order to examine this issue.

Finally, at present we cannot conclude that our findings are either state or trait markers of ADHD. A recent review suggests that atypical pupillary responses in autism spectrum disorder (ASD) might be associated with atypical attentional functions [35]. For example, elevation in tonic pupil size has also been found in children with ASD [36, 37] (but see [38]). On the other hand, findings on phasic pupil response in individuals with ASD are inconclusive [37–39]. As ADHD is the most common comorbidity in children with ASD [40, 41], it is possible that ADHD traits might play an important role in atypical pupillary responses in ASD. Conversely, only a minority of individuals with ADHD have an ASD diagnosis [42]. The question of whether both ASD and ADHD share an atypical alert trait or not, needs to be addressed in future research. Meanwhile, if these alterations in ADHD were restored by noradrenergic agents, such as methylphenidate or atomoxetine as previously reported [23], they would be useful as state markers for ADHD.

In conclusion, adults with ADHD demonstrated elevated tonic and attenuated phasic pupil dilations during an aCPT compared to TD adults. These observations may reflect dysfunction of the LC-NE system in adult ADHD, as well as the theoretically exploratory-biased alert system representative of ADHD. Future research will be necessary to clarify the underlying mechanisms of the atypical pupillary responses in individuals with ADHD.

## Materials and methods

### Participants

All of the experiments reported here were conducted in accordance with the ethical standards stipulated in the 1964 Declaration of Helsinki and approved by the Research Ethics Committee of Showa University. The participants consisted of 23 TD adults (10 men, age: 35.5 ± 1.7 years old) and 17 adults with ADHD (8 men, age: 31.7 ± 2.0 years old). Table 1 summarizes the demographic data of the participants, including chronological age, gender, full intelligence quotient (IQ), verbal IQ (VIQ), and performance IQ (PIQ) as defined by the Wechsler Adult Intelligence Scale-Third Edition, revised Japanese edition (WAIS-III). The individuals with ADHD were recruited from an outpatient clinic at Seiwa Hospital, Tokyo, Japan. ADHD was diagnosed using the Diagnostic and Statistical Manual of Mental Disorders, Fifth Edition (DSM-V) criteria by expert clinicians (including author TT). Twelve participants among the ADHD group were drug-naïve; the other five patients were treated with methylphenidate (MHP; average dose, 45-mg/day) or atomoxetine (ATX; average dose, 80-mg/day). They stopped taking their ADHD medications on the day of the experiment.

The participants in the TD group were selected to closely match the ADHD group in terms of their demographic features as noted above. There were no significant differences between the ADHD and TD groups in terms of age (unpaired t-test, p = .08), FIQ (*p* = .38), VIQ (*p* = .50), and PIQ (*p* = .19). As a screening tool, we used the Japanese version of the adult attention-deficit hyperactivity disorder (ADHD) Self-Report Scale (ASRS-J) [43]. The diagnosis was confirmed with a semi-structured interview (Assessment System for Individuals with ADHD: ASIA [44]) by author TT (an experienced psychiatrist for adults with ADHD). The ADHD group comprised patients with ADHD that were primarily inattentive (ADHD/I, N = 11) or combined inattentive/hyperactive (ADHD/C, N = 6).

The exclusion criteria for all participants were having a current major depressive or manic-depressive episode, a history of psychosis, FIQ < 80, a history of head injury with loss of consciousness, sensory-motor handicap, and neurological illness. No TD adult displayed clinically significant levels of ADHD symptomatology, as indexed by the ASRS-J. All participants had normal or corrected normal vision and normal hearing and provided written and informed consent before inclusion in the study. Participants were restricted from using caffeine and/or nicotine on the experimental day, as well as any medication that could affect eye movements [45]. They were also naïve to the task.

### Apparatus

The participants sat in a lit room facing a monitor screen, subtending 50.9 × 28.6 degrees of visual angle at 57 cm distance. A chin-rest was used to stabilize the participants' head position. Each task was preceded by a calibration procedure for which the participants were required to saccade to nine red dots that were presented sequentially in a square array. Stimuli were generated with the Psychophysics Toolbox routines [46, 47] for MATLAB (Version 2013b, MathWorks Ltd, http://www.mathworks.com/) and presented on a 23-inch LCD monitor (1920×1080 pixels at 60 Hz) driven by a PC (Windows 7).

During the whole experiment including the practice and test phases of the aCPT, the participant’s eye position and pupil diameter were monitored with a remote type eye tracker (model: TX300, Tobii Technology, Stockholm, Sweden) with a sampling rate of 300 Hz. This device enabled us to measure the pupil diameter of the participants over time, based on the distance between the eyes and the sensor.

### Procedure

#### Passive viewing

Before the auditory CPT task, the participants were instructed to maintain a steady fixation for 2 minutes on a black cross (0.87 cd/m^2^) subtending 0.5×0.5 degrees of visual angle. This was done to obtain baseline information before engaging the task.

#### Auditory CPT

The present study employed an acoustic consecutive performance test (aCPT [9]) that is schematically depicted in **Fig 1A**. The participants were instructed to respond as quickly and accurately as possible every time the target tone occurred and not to react to the nontarget tone. The target tone was 880 Hz and occurred with a probability of .20, whereas the nontarget tone was 800 Hz and occurred with a probability of .80. The participants delivered their responses using a keypad. The task continued without feedback. During the aCPT, the participants were instructed to maintain a steady fixation within an area indicated by a thin white line (62.9cd/m^2^) at the center of a gray background (25.65cd/m^2^). The area subtended 10 × 10 degrees of visual angle.

Each participant ran three blocks. Each block consisted of 100 trials and lasted about 8.5 minutes. Before starting the test blocks, the participants ran a practice block (20 trials) in which the target and nontarget tones were presented equally to familiarize the stimulus tones to the participants. Then either of the tones was presented for 250 ms followed by a random interstimulus interval (ISI) ranging from 3 to 5 seconds. After each block (8.5 min) a short break was given. It took about 40 min to complete the whole task.

### Data analysis

MATLAB was used to process and analyze pupil data. As pupil diameter data contained missing values as well as noise (e.g., the acute change of pupil diameter) induced by eye blinks, we first looked for missing data and eliminated irregular samples surrounding the gaps. Next, we estimated putative correct pupil diameter using linear interpolation [48]. Data from every trial of all participants was semi-automatically inspected after the interpolation, and all trials that had more than 50% of the data missing (due to unstable measurements) were excluded from the analysis. Two individuals in the TD group were eliminated from further investigations due to excessive loss of data. We used an average of 92.4% of the data (SD: 12.8) in the TD group, and 89.5% (SD: 20.6) in the ADHD group.

To estimate pupillary responses in the aCPT, we averaged pupillary fluctuations that were time-locked to the stimulus onset, separately per participant. To estimate tonic pupil diameter, we calculated an average pupil diameter in the period from -1 to 0 s before the stimulus presentation. Phasic pupil diameter was defined as a difference between pupil diameter maxima in a time window between after stimulus onset and 4 s after stimulus (target, nontarget) onset and tonic pupil diameter. We calculated phasic pupil dilation by subtracting the averaged tonic pupil diameter from the pupil diameter maxima after stimulus onset.

## Supporting information

S1 File Original data regarding pupillary responses during the passive viewing and the aCPT. (XLSX)

S2 File Original data regarding behavioral performance during the aCPT. (XLSX)
